# Supplementary material for: Germ cell connectivity enhances cell death in response to DNA damage in the Drosophila testis
Source: eLife. 2017 Aug 15;6:e27960. doi: 10.7554/eLife.27960 (PMC5577909; doi:10.7554/eLife.27960)
Supplement: Transparent reporting form [file elife-27960-transrepform.docx]

***eLife’s* transparent reporting form**

We encourage authors to provide detailed information *within their submission* to facilitate the interpretation and replication of experiments. If you have any questions, please contact us: [editorial@elifesciences.org](mailto:editorial@elifesciences.org).

**Sample-size estimation**

- You should state whether an appropriate sample size was computed when the study was being designed
- You should state the statistical method of sample size computation and any required assumptions
- If no explicit power analysis was used, you should describe how you decided what sample (replicate) size (number) to use

Please outline where this information can be found within the submission (e.g., page numbers or figure legends), or explain why this information doesn’t apply to your submission:

Exact sample sizes of each experiment performed with multiple biological replicates are provided in the corresponding figure legends, supplemental figure legends, or main text as appropriate. Biological replicate size was chosen based on number of animals that could be efficiently processed during the experimental procedure, typically n≥15.

**Replicates**

- You should report how often each experiment was performed
- You should include a definition of biological versus technical replication
- The data obtained should be provided and sufficient information should be provided to indicate the number of independent biological and/or technical replicates
- If you encountered any outliers, you should describe how these were handled
- Criteria for exclusion/inclusion of data should be clearly stated
- High-throughput sequence data should be uploaded before submission, with a private link for reviewers provided (these are available from both GEO and ArrayExpress)

Please outline where this information can be found within the submission (e.g., page numbers or figure legends), or explain why this information doesn’t apply to your submission:

Biological replicates were defined as identical repeats of the same tissue/genotype sample following the same experimental conditions, whereas technical replicates were experimental repeats using a single tissue sample (technical replicates = 1 for all experiments due to the nature of protocol). All experiments were performed with multiple biological replicates for each genotype (n≥3) and multiple animals used per replicate (listed in corresponding figure legends).

**Statistical reporting**

- Statistical analysis methods should be described and justified
- Raw data should be presented in figures whenever informative to do so (typically when N per group is less than 10)
- For each experiment, you should identify the statistical tests used, exact values of N, definitions of center, methods of multiple test correction, and dispersion and precision measures (e.g., mean, median, SD, SEM, confidence intervals; and, for the major substantive results, a measure of effect size (e.g., Pearson's r, Cohen's d)
- Report exact p-values wherever possible alongside the summary statistics and 95% confidence intervals. These should be reported for all key questions and not only when the p-value is less than 0.05.

For comparisons between experimental groups and sibling-matched controls (when applicable), unpaired t-test was used for comparison. Corresponding figure legends include the exact statistical tests used, definition of significance, and measures of dispersion and precision. For Supplementary Table 1, comparison of dataset distributions was done via unpaired Kolmogorov-Smirnov test. Raw numerical data included in “Source Data” document.

Please outline where this information can be found within the submission (e.g., page numbers or figure legends), or explain why this information doesn’t apply to your submission:

(For large datasets, or papers with a very large number of statistical tests, you may upload a single table file with tests, Ns, etc., with reference to page numbers in the manuscript.)

**Additional data files (“source data”)**

- We encourage you to upload relevant additional data files, such as numerical data that are represented as a graph in a figure, or as a summary table
- Where provided, these should be in the most useful format, and they can be uploaded as “Source data” files linked to a main figure or table
- Include model definition files including the full list of parameters used
- Include code used for data analysis (e.g., R, MatLab)
- Avoid stating that data files are “available upon request”

Please indicate the figures or tables for which source data files have been provided:

Fig 3-Numerical data for number of dying SG per 16-SG cyst in fusome mutants

Fig 4-Numerical data for number of dying SG per 16-SG cyst in *HtrA2/Omi* mutants

Fig 5-Numerical data for number of dying SG per 16-SG cyst in DNA damage response mutants
